# Practical Hints for the Sick-Room

**Published:** 1887-01-15

**Authors:** 


### Till the Doctor Comes.

[Inquiries and Communications for this column should be addressed, "Physician," care of the Editor of The Hospital.]

### VIII.—Practical Hints for the Sick-Room.

To give Cod Liver or Castor Oil.

Rinse out a wine glass with a little brandy, leaving a few drops at the bottom of the glass. Pour the dose of oil into the glass, and the spirit will roll the oil, so to speak, into a ball like the yolk of an egg, which can then be easily swallowed without any unpleasant taste. A little milk may be afterwards taken. Another good plan is to divide a lemon, squeezing the juice from each half into a separate tumbler. To the one add a wine-glassful of water, and sufficient sugar to make it palatable. In the other tumbler, beat up the dose of oil with the lemon juice, then add some sugar and a little water; stir this well up to the moment of swallowing, and after taking it, give the patient the previously prepared lemonade. Others recommend that the oil should be mixed with milk or coffee, or, better still, with a small basin of soup. It may be added that one teaspoonful of glycerine and two teaspoonfuls of castor oil make a most effectual dose.

To Apply Leeches.

Thoroughly clean the skin, and smear a little milk or porter over it. Place the leech or leeches in a glass, and invert over it. When gorged, they will usually drop off; if they do not, sprinkle a little salt over them. Never place them over a large vein. If more blood is desired, bathe the bites with hot water, or apply hot fomentations for a few minutes. If they bleed too freely, place a small thick pad of lint over them, and press firmly for some time.

To Apply a Blister.

Blistering fluid may be painted on the part, or a plaster may be employed. The former is cleaner and more simple. The latter should be bandaged on, not stuck down with adhesive plaster, which would be drawn up after a time as the blister rose, and cause much pain. It usually takes six to twelve hours for a blister to rise. When risen, the blister may be pricked to let out the fluid, and dressed with some simple ointment, or the thin raised skin may be removed altogether by cutting round its edge with scissors, and then the raw surface dressed with simple ointment, or, if it be desired to keep the blister open for some time, with savin ointment. If a blister does not rise after twelve hours, a poultice or hot water dressing will often cause it to do so.

To give an Enema.

Pass, first, plenty of hot water through the apparatus, with the object of warming it, and also of seeing that it works properly. The point must be oiled, and introduced into the lower part of the bowel, the patient lying on his left side. Enemas are given with two objects —(1) to relieve the bowels, when not less than 1½ to 2 pints of liquid must be used; (2) to supply nourishment to the patient, or to relieve diarrhœa, when small injections must be given of 2 to 3 oz.; —a small elastic bottle of that size is best for these cases. When large injections are used, they must be very gradually pumped up, stopping occasionally when they cause straining, till it has passed away. The second class of injections are used small, that they may be retained as long as possible. The following are some of the most commonly used injections:—

(1) Warm soapsuds and water.
 Warm gruel, very thin, strained through fine muslin, or it will clog the instrument.
 Castor oil, 2 or 3 oz. added to either of the above.

(2) Starch injection, 2 oz. of thin starch with 30 drops of laudanum. This is to relieve diarrhœa, and must only be given to a grown-up person.

Nourishing injections must be given under instructions from the doctor. They are now usually mixed with one of the numerous digestive extracts so much in vogue, which causes them to undergo a process of artificial digestion in the lower part of the bowel, and to be more readily taken into the system.

Subcutaneous injections should on no account be given, except after instructions and directions from the medical man.

